# Plasma interleukin 6 levels are associated with cardiac function after ST-elevation myocardial infarction

**DOI:** 10.1007/s00392-018-1387-z

**Published:** 2018-10-26

**Authors:** Hilde E. Groot, Lawien Al Ali, Iwan C. C. van der Horst, Remco A. J. Schurer, Hindrik W. van der Werf, Erik Lipsic, Dirk J. van Veldhuisen, Jacco C. Karper, Pim van der Harst

**Affiliations:** 1 Department of Cardiology, University of Groningen, University Medical Center Groningen, Hanzeplein 1, 9700 RB Groningen, The Netherlands; 2 Department of Critical Care, University of Groningen, University Medical Center Groningen, Groningen, The Netherlands

**Keywords:** Interleukin 6, Soluble interleukin 6 receptor, Soluble glycoprotein 130, ST-elevation myocardial infarction, Infarct size, Left ventricular ejection fraction

## Abstract

**Background and aims:**

Myocardial infarction triggers an inflammatory response involved in cardiac repair. We studied the association of the interleukin 6 (IL-6) cascade with infarct size and cardiac function after ST-elevation myocardial infarction (STEMI).

**Methods:**

In 369 STEMI patients IL-6, soluble IL-6 receptor (sIL-6R), and soluble glycoprotein (sgp) 130 were measured at baseline (hospital admission), 24 h, 2 weeks, 7 weeks, 4 months, and 1 year post-PCI and sIL-6R/IL-6 ratio was calculated. At 4 months, infarct size and left ventricular ejection fraction (LVEF) were assessed by magnetic resonance imaging. Diastolic function (*E*/*e*′) was determined by echocardiography.

**Results:**

Hospital admission levels for IL-6, sIL-6R, sgp 130 were 3.7 pg/ml (IQR 2.1–6.7 pg/ml), 51.6 ng/ml (IQR 37.3–69.0 ng/ml), and 332 ng/ml (IQR 280–399 ng/ml), respectively. 24 h after admission, IL-6 had increased threefold compared to baseline (*p* < 0.001) and returned below baseline (*p* < 0.001) 2 weeks after STEMI. sIL-6R and sgp130 levels at 24 h remained similar to baseline but were increased at 2 weeks (*p* < 0.001; *p* < 0.001, respectively). IL-6 and sIL-6R/IL-6 ratio at 24 h were independently associated with infarct size [*β* 5.4 (95% CI 3.3–7.5); *p* < 0.001, *β* − 4.0 (95% CI − 6.1 to − 1.9); *p* < 0.001, respectively]. Higher levels of IL-6 at 24 h were associated with lower LVEF [*β* − 4.2 (95% CI -6.7 to − 1.8); *p* = 0.001].

**Conclusions:**

Higher IL-6 and lower sIL-6R/IL-6 ratio early after presentation with STEMI are indicative for larger infarct size and decreased cardiac function at 4 months.

**Electronic supplementary material:**

The online version of this article (10.1007/s00392-018-1387-z) contains supplementary material, which is available to authorized users.

## Introduction

The past decades, a substantial amount of research has been dedicated to enhancing our understanding of the role of inflammation throughout the cardiovascular disease continuum. In myocardial infarction (MI) an intense inflammatory response that is essential for cardiac repair is observed, but which is also implicated in the pathogenesis of post-infarction remodeling and heart failure [5]. An intriguing diversity of potentially targetable mechanisms in the inflammatory cascade has been discovered [2, 3, 8, 14, 15, 25, 29, 32]. Recently, the Canakinumab Anti-inflammatory Thrombosis Outcome Study (CANTOS) demonstrated that anti-inflammatory therapy targeting the interleukin-1β (IL-1β) pathway with the monoclonal antibody canakinumab leads to a significantly lower rate of recurrent cardiovascular events in patients with previous MI [21].

IL-1β is known to drive the IL-6 signaling pathway, and therefore, this pathway could be another potential therapeutic target in the treatment of coronary artery disease [20]. IL-6 itself is conserved to be a central hub in cardiometabolic signaling and can be produced by many different cells regulating the acute-phase response [16, 20]. IL-6 can bind to the membrane bound IL-6 receptor (IL-6R) and the soluble form of IL-6R (sIL-6R). It contributes to atherosclerotic plaque development and destabilization [30]. Furthermore, mendelian randomization studies of the IL-6R provided evidence for a causal mechanism of IL-6 signaling in the development of coronary artery disease [9, 10]. Downstream signaling of the IL-6R also is thought to play a role in the regulation of cardiomyocyte loss, cardiac hypertrophy, and loss of cardiac function [4]. Studies in patients presenting with an acute coronary syndrome also suggested that IL-6 is associated with ischemia–reperfusion myocardial injury and mortality [24, 31].

The humanized anti-IL-6R antibody tocilizumab showed to block IL-6 binding on the receptor, and to be effective and generally well tolerated in patients with autoimmune disorders. In patients with non-ST-elevation MI treated with percutaneous coronary intervention (PCI) tocilizumab treatment was associated with a reduced inflammatory response and lower troponin T levels [12].

Whether the IL-6 pathway can be linked to MI size and cardiac function is unknown. We hypothesized that levels of the IL-6 cascade components during admission for MI are associated with infarct size and cardiac function.

## Methods

### Study population and design

We included all patients participating in the GIPS-III trial. This trial was designed to evaluate the effect of metformin treatment on preservation of left ventricular function in STEMI patients without diabetes and to establish a biobank. Details on the design of the GIPS-III trial have been reported previously [13]. In brief, all patients admitted to the University Medical Center Groningen between January 1st, 2011, and May 26th 2013, via the STEMI protocol were considered eligible for the trial. Inclusion criteria were age older than 18 years, the presence of STEMI, and primary PCI with implantation of at least 1 stent with a diameter of at least 3 mm resulting in TIMI flow grade 2 or 3 post PCI. Major exclusion criteria were previous myocardial infarction, known diabetes, the need for coronary artery bypass graft surgery, severe renal dysfunction, and standard contraindications for magnetic resonance imaging (MRI) [14]. The study protocol of the GIPS-III trial was in accordance with the Declaration of Helsinki and was approved by the local ethics committee (Groningen, the Netherlands) and national regulatory authorities. Informed consent was obtained for inclusion of the patients.

### Data collection

On admission, standard laboratory assessment was performed and standard physical examination parameters were measured according to protocol. Patients were seen in the outpatient clinic 2 weeks, 7 weeks, 4 months, and 1 year after discharge.

During hospitalization, blood was sampled at baseline (initial admission) and at 3, 6, 9, 12, and 24 h after PCI to monitor values of cardiac enzymes and high sensitive troponin. Less frequently during hospitalization and at every visit to the outpatient clinic hemoglobin, leucocytes (including neutrophils, lymphocytes), platelets, glucose, hs-CRP and N-terminal pro B-type natriuretic peptide (NT-proBNP) were determined. Furthermore, during PCI, 24 h after PCI, and at every visit to the outpatient clinic, blood samples for additional analyses were collected [13]. LVEF was measured by MRI 4 months after infarction. Imaging was performed on a 3.0 T whole-body MRI scanner (Achieva; Philips) using a phased array cardiac receiver coil. Electrocardiogram-gated cine steady state, free precession magnetic resonance images were acquired during repeated breath holds in contiguous short-axis slices of 1 cm covering the entire left ventricle. The endocardial borders were outlined in end-systolic and end-diastolic images. Left ventricular end-systolic volumes and left ventricular end-diastolic volumes were calculated using the summation of slice method multiplied by slice thickness. An independent core laboratory (Image Analysis Center, VU University Medical Center, Amsterdam, the Netherlands) evaluated the MRI scans and assessed the primary efficacy measure, blinded for treatment allocation and clinical patient data. According to the guidelines, LVEF ≥ 50% was considered as normal, LVEF < 40% was considered as reduced, LVEF between 40–49% was considered as ‘mid-range’ [18]. Additionally, transthoracic echocardiograms were performed in left lateral decubitus position using a Vivid 7 echo system (General Electric, Horton, Norway) at 4 months. The echocardiographic data from these echocardiograms were digitally stored in DICOM format and analyzed off-line by an independent core lab (Groningen Imaging Core Laboratory, Groningen, the Netherlands) that was blinded to treatment allocation and clinical information. *E*/*e*′ was used as a measure of diastolic function. Mitral valve early filling flow (*E*) and early diastolic tissue velocities (*e*′) from both the septal and lateral wall were measured in accordance with current guidelines [17]. Mean *e*′ was calculated as (*e*′ septal + *e*′ lateral)/2. *E*/*e*′ was determined as *E*/mean *e*′ and was deemed abnormal if ≥ 13. The incidence of major adverse cardiac events (MACE; the combined endpoint of death, reinfarction, or target-lesion revascularization) was recorded until 4 months [13].

### IL-6, sIL-6R, and sgp130 measurements

To determine the IL-6 levels, we performed enzyme-linked immunosorbent assay (ELISA) in serum, using human IL-6 DuoSet ELISA (R&D, cat. no. DY206). To determine the sIL-6R levels, we used human IL-6R DuoSet ELISA (R&D, cat. no. DY227), and for determining sgp130, we used human sgp130 DuoSet ELISA (R&D, cat. no. DY228). Samples were randomly analyzed. In our laboratory, the inter-assay coefficients of variation (CV) for IL-6, sIL-6R, and sgp130 were, respectively, 6.3%, 11.6%, and 5.9%. The cytokines were measured in duplicate and the detectable limits for IL-6, sIL-6R, and sgp130 were, respectively, 0.4 pg/ml, 1.4 ng/ml, 0.1 ng/ml. Since IL-6 binds to IL-6R we also calculated the IL-6R/IL-6 ratio.

### Myocardial blush grade

Myocardial blush grade (MBG) represents an angiographic measurement of myocardial perfusion [7]. It reflects a myocardial response to ischemic injury and reperfusion. MBG was categorized as follows: 0 = no myocardial blush, or contrast density; 1 = minimal myocardial blush; 2 = moderate myocardial blush but less than that obtained during angiography of a contralateral or ipsilateral non-infarct-related coronary artery; 3 = normal myocardial blush comparable to that obtained during angiography of a contralateral or ipsilateral non-infarct-related coronary artery [28]. The patients were categorized as having normal (MBG 3) versus impaired (MBG 0–2) reperfusion. Coronary angiograms were analyzed by two physicians blinded to clinical data.

### Statistical analysis

Continuous variables were summarized as mean ± standard deviation if normally distributed or median and interquartile range if skewed. Discrete variables were presented as frequencies and percentages. To compare groups, we used Student’s *t* test for normally distributed continuous variables, Mann–Whitney *U* test for skewed continuous variables, Chi-square and Fisher’s exact test for categorical variables. We used mixed model analysis to analyze biomarker levels between patient groups over time. We modeled IL-6, sIL-6R, sIL-6R/IL-6 ratio, and sgp130 using a random intercepts regression model in terms of MBG, LVEF, and *E*/*e*′, and time points. Regarding IL-6, sIL-6R, sIL-6R/IL-6 ratio, and sgp130, we created quartiles to use them in regression analysis (using the first quartile as reference). Relevant variables (Table 1) were assessed as potential confounders. Variables with a *p* < 0.2 in univariate analysis were included in the multivariate linear regression model. In accordance with Benjamin et al., a two-tailed *p* value of < 0.005 was considered significant. *p* value between 0.05 and 0.005 was considered suggestive [1]. Statistical analyses were performed with Stata version 14.0 (StataCorp). Figures were created with GraphPad Prism version 7.02 for Windows.

**Table 1:** Baseline characteristics

Characteristics	Total (*n* = 369)
Age, mean (SD), years	58.8 ± 11.6
Female sex—no. (%)	95 (25)
BMI, mean (SD), kg/m^2^	27.0 ± 3.8
Cardiovascular related history—no. (%)	
Hypertension	112 (30)
Dyslipidemia	239 (63)
Current smoking	209 (55)
Stroke	3 (0.8)
Peripheral artery disease	0
Previous PCI	4 (1.1)
Blood pressure, mean (SD), mmHg	
Systolic	134 ± 23
Diastolic	84 ± 15
Heart rate, mean (SD), beats/min	76 ± 16
Ischemia time, median (IQR), min	161 (109–250)
Single vessel disease—no. (%)	258 (68)
Infarct-related artery TIMI flow—no. (%)	
Preintervention grade	
0	208 (55)
1	27 (7.1)
2	66 (17)
3	78 (21)
Post-intervention grade	
2	34 (9.0)
3	345 (91)
Myocardial blush grade	
0	10 (2.6)
1	29 (7.7)
2	74 (20)
3	263 (69)
Laboratory values at admission	
CK, median (IQR), U/l	129 (83–210)
Myocardial band of CK, median (IQR), U/l	16 (13–25)
Troponin, median (IQR), ng/l	50 (23–136)
Creatinine, median (IQR), umol/l	72 (62–82)
NT-proBNP, median (IQR), ng/l	81 (40–200)
Glucose median (IQR), mmol/l	8.2 (7.0–9.6)
HbA1c, median (IQR), %	5.8 (5.6–6.0)
Blood count and biochemistry	
Leucocytes (10e9/l)	11 (9–14)
Thrombocytes (10e9/l)	234 (204–269)
Neutrophils (10e9/l)	8 (5–10)
Lymphocytes (10e9/l)	2 (1–3)
N/L ratio	3.63 (2.39–6.07)
Hs-CRP (mg/l)	2.1 (1–4.2)

Data are expressed as mean ± standard deviation (SD), median [interquartile range (IQR)], or as number (%)

*BMI* Body Mass Index, *TIMI* thrombolysis in myocardial infarction, *CK* creatine kinase, *NT-proBNP* N-terminal pro brain natriuretic peptide, *HbA1c* glycated hemoglobin, *N/L* neutrophil/lymphocyte

## Results

### Baseline characteristics

Baseline characteristics are presented in Table 1 and in Supplementary Tables 1, 2 and 3 according to quartiles of IL-6, sIL-6R and sgp130. The average age at presentation was 59 years (± 12 year) and 25% of the population was female. One-third had hypertension, more than half smoked, and two-third had dyslipidemia. The median ischemic time was 161 min (IQR 109–250 min). Of the total 379 patients participating in the GIPS-III trial, we had plasma samples available of 369 patients. The exact number of samples which was available for the measurements of IL-6, sIL-6R and sgp130 at the different time points is presented in Supplementary Table 4. The range varied between 369 samples at baseline and 252 samples at 1 year follow-up.

### Interleukin 6, soluble interleukin 6 receptor, and soluble glycoprotein 130

**Fig. 1:**
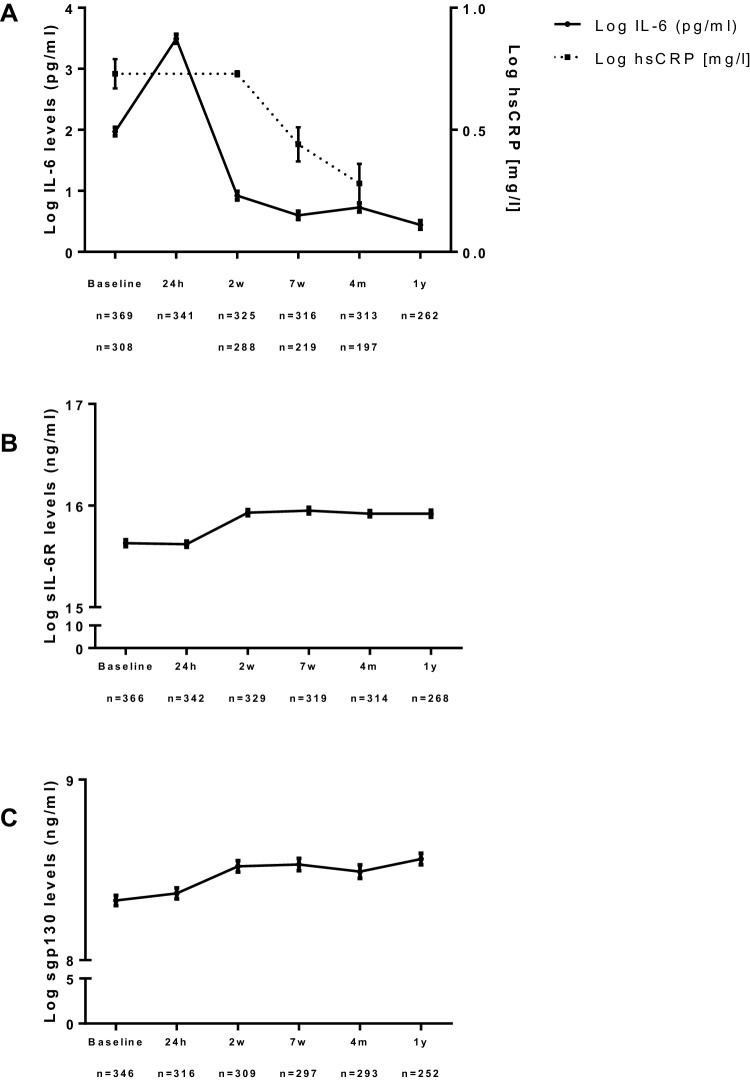
Log IL-6 and log hs-CRP (**a**), log sIL-6R (**b**), and log sgp130 levels (**c**). Levels are depicted in mean and SEM. IL-6 levels at baseline significantly differed from all consecutive time points (*p* < 0.001). hs-CRP levels at baseline significantly differed from hs-CRP at 7 weeks and 4 months (*p* < 0.001). sIL-6R and sgp130 levels at baseline significantly differed from sIL-6R at 2 weeks, 7 weeks, 4 months, and 1 year (*p* < 0.001)

Median baseline IL-6 level was 3.7 pg/ml (IQR 2.1–6.7 pg/ml) and after 24 h it was increased a threefold to 10.3 pg/ml (IQR 5.8–19.8 pg/ml) (*p* < 0.001) and subsequently decreased to 1.8 pg/ml (IQR 1.1–2.9; *p* < 0.001) at 2 weeks to remain stable (Fig. 1a). The median sIL-6R at baseline was 51.6 ng/ml (IQR 37.3–69.0 ng/ml), did not change at 24 h, but increased somewhat after 2 weeks to 62.9 ng/ml (IQR 47.8–81.6 ng/ml; *p* < 0.001) and remained stable thereafter (Fig. 1b). Baseline sgp130 was 332 ng/ml (IQR 280–399 ng/ml) and showed a comparable trend as sIL-6R levels (Fig. 1c).

Baseline ratio between sIL-6R and IL-6 was 13,651 (IQR 6780–26,102), decreased a 2.8-fold to 4856 (IQR 2350–8382; *p* < 0.001) after 24 h and increased 8.7-fold to 36,843 (IQR 20,078–62,352; *p* < 0.001) at 2 weeks (Supplementary Figure 1A). The sIL-6R/IL-6 ratio at 24 h was ~ 10 times smaller compared to the sIL-6R/IL-6 ratio at 1 year (Supplementary Figure 1B).

Hs-CRP levels decreased significantly between 2 and 7 weeks post-PCI (*p* < 0.001) (Fig. 1).

### Interleukin 6 and reperfusion

Next, we studied IL-6 levels in relation to reperfusion grade. IL-6 levels were suggestively higher in a patient with impaired (MBG 0–2) compared to normal (MBG 3) reperfusion grade (*p* = 0.019; Supplementary Figure 2A). sIL-6R levels did not differ between groups (Supplementary Figure 2B) but the sIL-6R/IL-6 ratio was lower in patients with impaired reperfusion (*p* = 0.003; Supplementary Figure 2C). Sgp130 levels were also comparable between groups (data not shown).

### Differences in levels of the IL-6 cascade between normal and reduced cardiac function

**Fig. 2:**
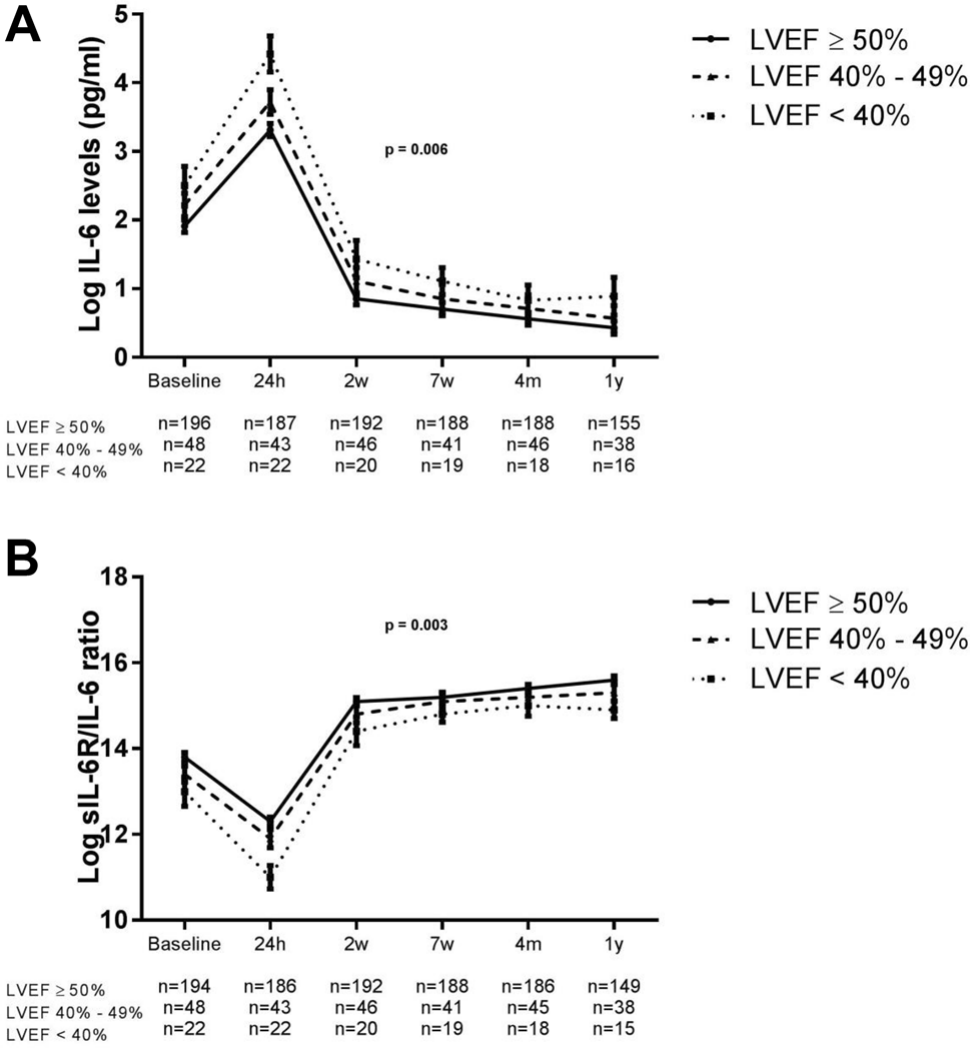
Log IL-6 (**a**), log sIL-6R/IL-6 (**b**) levels in STEMI patients with LVEF ≥ 50%, LVEF 40–49%, LVEF < 40%. Levels are depicted in mean and SEM. P values for trend are shown. IL-6 and sIL-6R/IL-6 ratio levels at baseline significantly differed from all consecutive time points (*p* < 0.001)

We compared IL-6 levels in patients categorized for their LVEF at 4 months. IL-6 levels were significantly higher in patients with reduced LVEF compared to the other categories (*p* = 0.006; Fig. 2a). Also the sIL-6R/IL-6 ratio was significantly lower in patients with reduced LVEF (*p* = 0.003; Fig. 2b). sIL-6R, sgp 130, and hs-CRP levels did not differ between categories (data not shown).

When studying the IL-6 cascade markers in relation to diastolic function, indicated by *E*/*e*′, IL-6 levels were suggestively higher in patients with abnormal *E*/*e*′ (*p* = 0.013) (Supplementary Figure 3A). sIL-6R/IL-6 ratio was significantly lower in patients with abnormal *E*/*e*′ (*p* = 0.005) (Supplementary Figure 3B). Similar to the biomarkers and LVEF, sIL-6R and sgp 130 levels did not differ between groups (data not shown).

### Associations between covariates and infarct size, cardiac function, and cardiac markers

Univariate associations between covariates and infarct size, LVEF or *E*/*e*′ are given in Table 2. After adjustments for relevant covariates, Q4 of IL-6 measured at baseline, compared to the lowest quartile (Q1), was still suggestively associated with infarct size with a coefficient of 2.87 (95% CI 0.74–5.00; *p* = 0.008; adj. *R*^2^ 0.19) (Supplementary Figure 4) and Q4 of sgp130 was suggestively associated with lower *E*/*e*′ [*β* − 0.82 (95% CI − 1.54 to − 0.11); *p* = 0.024; adj. *R*^2^ 0.19].

**Table 2:** Univariate associations between covariates and infarct size, LVEF, and *E*/*e*′ ratio measured at 4 months

	Infarct size		Left ventricular ejection fraction		*E*/*e*′ ratio	
	Coefficient (95% CI)	*p* value	Coefficient (95% CI)	*p* value	Coefficient (95% CI)	*p* value
Sex, female	− 0.35 (− 2.7 to 2.0)	0.77	1.6 (− 0.91 to 4.0)	0.22	2.0 (1.3 to 2.6)	< 0.001
Age, years	0.03 (− 0.06 to 0.11)	0.54	− 0.01 (− 0.10 to 0.74)	0.76	0.06 (0.04 to 0.09)	< 0.001
BMI, kg/m^2^	− 0.24 (− 0.53 to 0.04)	0.097	0.16 (− 0.13 to 0.45)	0.27	0.08 (− 0.00 to 0.16)	0.059
Heart rate, bpm	0.01 (− 0.05 to 0.08)	0.66	− 0.02 (− 0.09 to 0.04)	0.45	0.01 (− 0.00 to 0.03)	0.20
Hypertension	− 0.73 (− 2.9 to 1.5)	0.51	0.42 (− 1.8 to 2.7)	0.71	1.7 (1.0 to 2.4)	< 0.001
Hypercholesterolemia	1.4 (− 0.67 to 3.4)	0.19	− 1.6 (− 3.7 to 0.45)	0.13	0.14 (− 0.49 to 0.78)	0.66
Cerebrovascular accident	− 5.6 (− 21.3 to 10.0)	0.48	7.4 (− 9.3 to 24.1)	0.38	0.67 (− 2.6 to 3.9)	0.68
PTCA in medical history	3.4 (− 5.7 to 12.4)	0.46	− 1.7 (− 10.2 to 6.7)	0.69	− 1.2 (− 4.4 to 2.0)	0.47
Smoking	− 1.1 (− 3.1 to 0.86)	0.27	0.21 (− 1.8 to 2.2)	0.84	− 0.75 (− 1.4 to − 0.13)	0.018
TIMI (pre-intervention)	− 2.6 (− 3.3 to − 1.8)	< 0.001	1.5 (0.68 to 2.3)	< 0.001	− 0.79 (− 0.33 to 0.17)	0.54
TIMI (post-intervention)	− 3.8 (− 7.8 to 0.18)	0.06	3.3 (− 0.85 to 7.5)	0.12	− 0.26 (− 1.4 to 0.88)	0.66
MBG	− 1.9 (− 3.3 to − 0.49)	0.008	2.1 (0.67 to 3.5)	0.004	− 0.14 (− 0.57 to 0.30)	0.54
Ischemic time, min	0.00 (− 0.00 to 0.01)	0.51	− 0.01 (− 0.01 to 0.00)	0.15	0.00 (0.00 to 0.00)	0.60
IL-6 levels at baseline (Q4 vs Q1)	3.3 (1.0 to 5.6)	0.005	− 2.1 (− 4.5 to 0.23)	0.077	0.53 (− 0.23 to 1.3)	0.17
IL-6 levels at 24 h (Q4 vs Q1)	6.0 (3.7 to 8.2)	< 0.001	− 4.6 (− 7.0 to − 2.1)	< 0.001	1.2 (0.46 to 2.0)	0.002
sIL-6R levels at baseline (Q4 vs Q1)	− 1.5 (− 3.8 to 0.79)	0.20	0.07 (− 2.3 to 2.5)	0.96	− 0.10 (− 0.83 to 0.64)	0.79
sIL-6R levels at 24 h (Q4 vs Q1)	− 3.13 (− 5.36 to − 0.89)	< 0.001	1.5 (− 0.96 to 3.9)	0.23	− 0.28 (− 1.1 to 0.49)	0.47
Ratio sIL-6R/IL-6 at baseline (Q4 vs Q1)	− 2.8 (− 5.1 to − 0.46)	0.019	2.0 (− 0.43 to 4.4)	0.11	− 0.77 (− 1.5 to − 0.48)	0.037
Ratio sIL-6R/IL-6 at 24 h (Q4 vs Q1)	− 4.5 (− 6.8 to − 2.3)	< 0.001	3.3 (0.84 to 5.7)	0.008	− 0.90 (− 1.7 to − 0.14)	0.021
Sgp130 at baseline (Q4 vs Q1)	− 0.65 (− 3.2 to 1.9)	0.61	0.91 (− 1.7 to 3.5)	0.49	− 0.93 (− 1.7 to − 0.15)	0.020
Sgp130 at 24 h (Q4 vs Q1)	− 2.6 (− 5.2 to − 0.08)	0.043	3.1 (0.34 to 5.8)	0.028	− 0.35 (− 1.2 to 0.47)	0.40

*95% CI* 95% confidence interval, *BMI* Body Mass Index, *PTCA* percutaneous transluminal coronary angioplasty, *TIMI* thrombolysis in myocardial infarction, *MBG* myocardial blush grade, *IL-6* interleukin 6, *sIL-6R* soluble interleukin 6 receptor, *sgp130* soluble glycoprotein 130, *Q1* lowest quartile, *Q4* highest quartile

When analyzing for the association between biomarker levels at 24 h and infarct size, LVEF or *E*/*e*′, the Q4s of IL-6 and sIL-6R/IL-6 ratio vs the lowest quartile were significantly associated with infarct size, also after adjusting for covariates [*β* 5.41 (95% CI 3.33–7.48); *p* < 0.001; adj. *R*^2^ 0.23, *β* − 4.00 (95% CI − 6.11 to − 1.89); *p* < 0.001; adj. *R*^2^ 0.20, respectively]. The Q4 of sIL-6R at 24 was suggestively associated with smaller infarct size [*β* − 2.45 (95% CI − 4.56 to − 0.35); *p* = 0.023; adj. *R*^2^ 0.17].

Looking at LVEF, the Q4 of IL-6 at 24 h remained significantly associated after adjustment [*β* − 4.24 (95% CI − 6.67 to − 1.80); *p* = 0.001; adj. *R*^2^ 0.08]. The Q4 of sIL-6R/IL-6 ratio at 24 h was suggestively associated with higher LVEF [*β* 2.63 (95% CI 0.18–5.08); *p* = 0.035; adj. *R*^2^ 0.06].

**Fig. 3:**
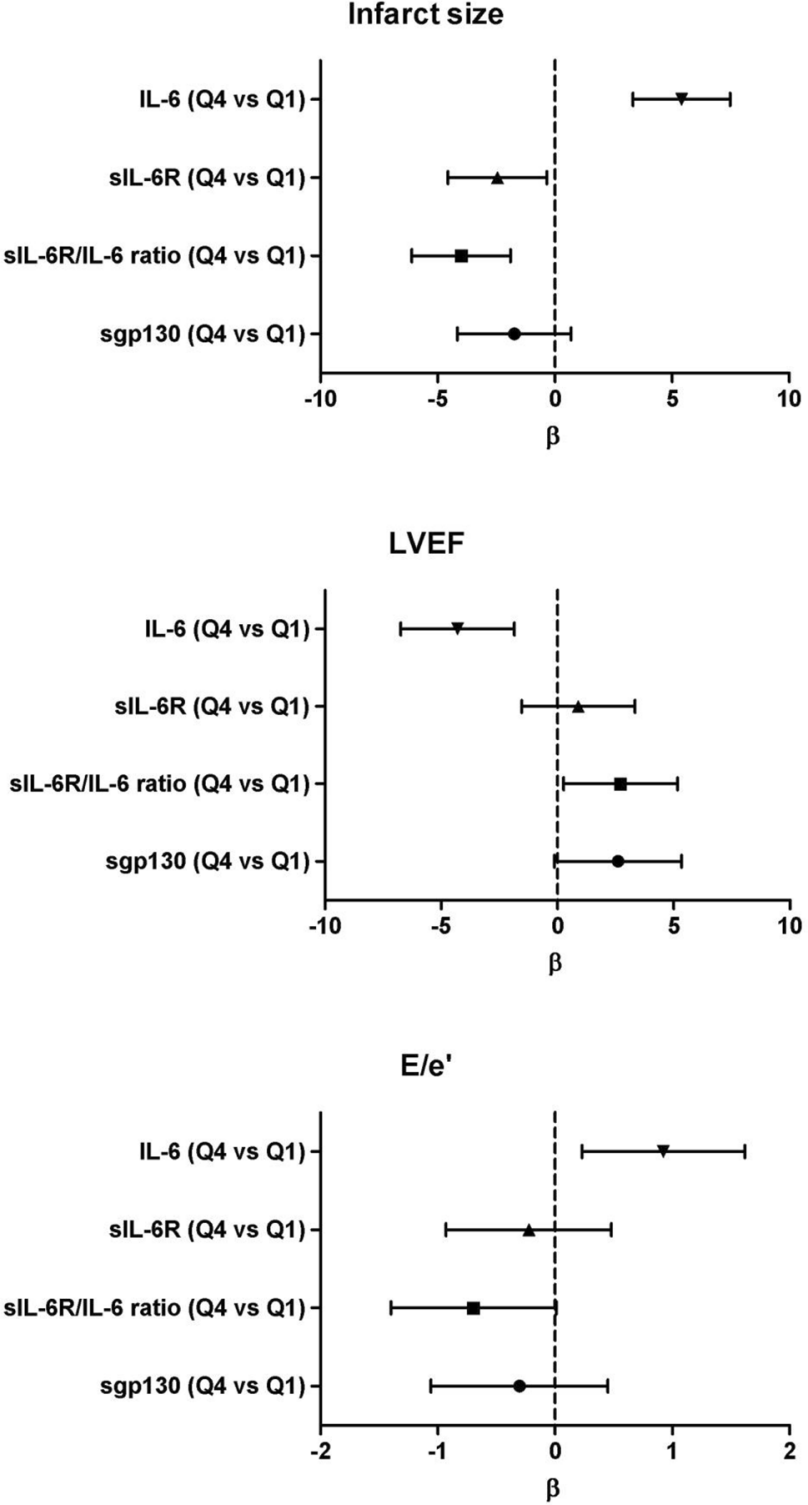
Associations between members of the interleukin-6 signaling cascade measured in STEMI patients at 24 h and infarct size, LVEF, and *E*/*e*′ measured at 4 months, depicted as *β* and 95% CIs obtained from linear regression models. *95% CI* 95% Confidence Interval, *IL-6* interleukin 6, *sIL*-*6R* soluble interleukin 6 receptor, *sgp130* soluble glycoprotein 130, *Q1* lowest quartile, *Q4* highest quartile, *STEMI* ST-elevation myocardial infarction, *LVEF* left ventricular ejection fraction. Multivariate analysis on infarct size: adjusted for age, sex, BMI, hypercholesterolemia, TIMI (pre- and post-intervention), MBG. Multivariate analysis on LVEF: adjusted for age, sex, hypercholesterolemia, TIMI (pre- and post-intervention), ischemic time. Multivariate analysis on *E*/*e*′: adjusted for age, sex BMI, heart rate, hypertension, smoking

The Q4 of IL-6 at 24 h was suggestively associated with diastolic function as indicated by the *E*/*e*′ [*β* 0.92 (95% CI 0.23–1.62; *p* = 0.010; adj. *R*^2^ 0.20)] (Fig. 3). All associations persisted after correction for hs-CRP.

There were no associations of members of the IL-6 cascade and diastolic function within the different LVEF groups (data not shown). Furthermore, IL-6 at baseline was significantly associated with troponin T, CK, CK-MB and NT-proBNP (0.19, *p* < 0.001; 0.19, *p* < 0.001; 0.22, *p* < 0.001; 0.22, *p* < 0.001, respectively) (Supplementary Table 5).

### Outcomes

The number of clinical events in the overall population was low, mortality rate at 2 years was 1.1%, reinfarction rate at 2 years was 3.4%. There were no associations between the components of the IL-6 system and reinfarction or mortality (data not provided).

## Discussion

In patients presenting with a first STEMI treated with primary PCI, the temporal course of the IL-6 cascade components is associated with infarct size and cardiac function. IL-6 levels are increased during the first 2 weeks and reach a steady state afterward. Furthermore, sIL-6R and sgp130 levels are decreased during the first 2 weeks, after which they reach a stable level, similar to IL-6. Most importantly IL-6 and sIL-6R/IL-6 ratio at 24 h are associated with infarct size and cardiac function measured at 4 months. sIL-6R and gp130 alone are not associated with these outcomes, although higher levels of sgp130 are suggestively associated with lower *E*/*e*′.

### Inflammation after myocardial infarction

Several pre-clinical studies show the benefits of intervening in the inflammatory cascade during/after MI [4]. So far, beneficial effects of intervening in the IL-6 cascade have not been observed in clinical studies, probably because of the pleiotropic characteristics of IL-6.

Targeting the IL-1β pathway with canakinumab led to a lower rate of recurrent cardiovascular events than placebo [21]. Whether anti-inflammatory therapy is also protective in STEMI patients, is not elucidated yet. In contrast to chronic inflammation, which is considered to be harmful, the first acute inflammatory response in STEMI patients might even be cardioprotective facilitating repair of the infarction [4]. However, if this process continues and becomes chronic, this could cause excessive damage and fibrosis development eventually leading to loss of cardiac function [4].

### Temporal course of IL-6, sIL-6R, and sgp130

We studied the temporal course of IL-6, sIL-6R, and sgp130 during a long time span and their association with infarct size, LVEF and *E*/*e*′ [12, 22, 23, 31]. IL-6, sIL-6R as well as sgp130 reach a stable state around approximately 2 weeks after MI. Our findings are expanding the time horizon of these biomarker trajectories in comparison to earlier studies only evaluating the acute phase. We also present a broader picture of the IL-6 pathway by also measuring sIL-6R and sgp130, and calculating the ratio between sIL-6R and IL-6 [11, 19, 27]. Finally, we provide the first link of IL-6 pathway to normal/impaired reperfusion, systolic function and diastolic function, adding to the increasing level of evidence linking interleukin pathways in MI and the future development of decreased cardiac function and the risk of heart failure [6, 26].

### IL-6, infarct size, and cardiac function

In agreement with previous research, we observed associations between the IL-6, sIL-6R, sgp130 and infarct size and cardiac function [23]. We did not observe any associations between sgp130 and LVEF. This difference might be explained by differences in method and time course of LVEF measurements. Furthermore, the median time interval from symptoms to blood sampling was shorter compared to our study [23]. This is a small difference, although these hours could make a difference in case of the acute and sub-acute phase of the inflammatory response. In agreement with previous research, we did not observe a significant association between sIL-6R and LVEF either [23]. We could not confirm previous associations between the IL-6 cascade and long-term outcomes [22, 33]. This could be explained by a difference in sample size, since 989 and 525 STEMI patients were included. Since both studies observed an association between IL-6 signaling and outcome in both the general population and STEMI patients, this could be a promising marker of cardiovascular risk and even be used to select patients for anti-inflammatory therapy.

### Limitations

Some limitations should be taken into consideration. First, the GIPS III study recruited non-diabetic patients presenting with a first STEMI and as a consequence of rapid primary PCI the myocardial infarct size was limited and their systolic LV function well preserved. Second, we report associations and cannot draw conclusions about causality. Finally, our study was not powered to translate IL-6, sIL-6R, and sgp130 levels to clinical decision-making, and therefore, our results are not directly applicable to the clinical arena in terms of relevance for patient management but should be considered in the light of other available data.

### Future perspectives

Our study supports further research into targeting the IL-6 cascade in the treatment of acute myocardial infarction. One earlier study observed attenuation of the inflammatory response and troponin T release by tocilizumab. The associations we observed between IL-6, infarct size and cardiac function are additive to these results [12]. Furthermore, they suggested investigating the time of administration of tocilizumab regarding the inflammatory response.

### Conclusion

Members of the IL-6 cascade measured at 24 h after myocardial infarction are indicative for larger infarct size and decreased cardiac function measured at 4 months. These results support the concept of early intervention in the inflammatory cascade to prevent the heart from myocardial damage.

## Electronic supplementary material

Below is the link to the electronic supplementary material.

Supplementary material 1 (DOCX 41 KB)

Supplementary Figure 1. Log sIL-6R/IL-6 ratio (mean and SD) (A). We divided log sIL-6R levels by IL-6 levels to be able to show a clear and understandable figure. Reduction factor of log sIL-6R/IL-6 ratio using log sIL-6R/IL-6 ratio at 1 year as a reference (B). Supplementary Figure 2. Log hs-CRP levels in the total STEMI population (A) and in STEMI patients with LVEF ≥ 50%, LVEF 40 – 49%, LVEF < 40%. Levels are depicted in mean and SEM. P values for trend are shown. Supplementary Figure 3. Log IL-6 (A), log sIL-6R (B), and log sIL-6R/IL-6 ratio (C) levels in STEMI patients with normal (MBG 3) and impaired reperfusion (MBG 0-2). Levels are depicted in mean and SEM (IL-6 and sIL-6R) or mean and SD (sIL-6R/IL-6 ratio). *p* values for trend are shown. Supplementary Figure 4. Log IL-6 (A), log sIL-6R/IL-6 (B) levels in STEMI patients with normal E/e’ (<13) and elevated E/e’ (≥ 13). Levels are depicted in mean and SEM. *p* values for trend are shown. Supplementary Figure 5. Associations between members of the interleukin-6 signaling cascade measured in STEMI patients at baseline and infarct size, LVEF, and E/eˈ ratio measured at 4 months, depicted as β and 95% CIs obtained from linear regression models. 95% CI = 95% Confidence Interval; IL-6 = interleukin 6; sIL-6R = soluble interleukin 6 receptor; sgp130 = soluble glycoprotein 130; Q1 = lowest quartile; Q4 = highest quartile; STEMI = ST-elevation myocardial infarction; LVEF = left ventricular ejection fraction. Multivariate analysis on infarct size: adjusted for age, sex, BMI, hypercholesterolemia, TIMI (pre- and post-intervention), MBG. Multivariate analysis on LVEF: adjusted for age, sex, hypercholesterolemia, TIMI (pre- and post-intervention), ischemic time. Multivariate analysis on E/eˈ: adjusted for age, sex, BMI, heart rate, hypertension, smoking (PDF 743 KB)
