# Supplementary material for: Plasma interleukin 6 levels are associated with cardiac function after ST-elevation myocardial infarction
Source: Clin Res Cardiol. 2018 Oct 26;108(6):612–21. doi: 10.1007/s00392-018-1387-z (PMC6529378; doi:10.1007/s00392-018-1387-z)
Supplement: Supplementary file 1 — Supplementary material 1 (DOCX 41 KB) [file 392_2018_1387_MOESM1_ESM.docx]

Supplementary table 1. Baseline characteristics per IL-6 quartile

| **Characteristics** | **Q1 (n = 93)** | **Q2 (n = 92)** | **Q3 (n = 92)** | **Q4 (n = 92)** | **P value** |
| --- | --- | --- | --- | --- | --- |
| IL-6 levels (pg/ml) | 1.5 (1.1 - 1.8) | 2.8 (2.4 - 3.2) | 4.9 (4.1 - 5.6) | 10.3 (7.9 - 17.1) | <0.001 |
| Age, mean (SD), years | 57.4 ± 10.6 | 58.4 ± 11.4 | 58.6 ± 12.2 | 60.9 ± 11.9 | 0.22 |
| Female sex – No. (%) | 24 (26) | 19 (21) | 26 (28) | 24 (26) | 0.68 |
| BMI, mean (SD), kg/m^2^ | 27.0 ± 3.9 | 27.0 ± 3.7 | 27.0 ± 4.4 | 26.7 ± 3.3 | 0.69 |
| Cardiovascular related history – No. (%) |  |  |  |  |  |
| Hypertension | 25 (27) | 24 (26) | 24 (26) | 35 (38) | 0.21 |
| Dyslipidemia | 62 (67) | 54 (59) | 51 (55) | 63 (68) | 0.20 |
| Current smoking | 58 (62) | 47 (51) | 50 (54) | 50 (54) | 0.46 |
| Stroke | 1 (1) | 1 (1) | 1 (1) | 0 (0) | 0.80 |
| Peripheral artery disease | 0 (0) | 0 (0) | 0 (0) | 0 (0) | 1.00 |
| Previous PCI | 1 (1.1) | 1 (1.1) | 1 (1.1) | 1 (1.1) | 1.00 |
| Blood pressure, mean (SD) mmHg |  |  |  |  |  |
| Systolic | 133 ± 22 | 137 ± 24 | 134 ± 24 | 133 ± 24 | 0.57 |
| Diastolic | 82 ± 14 | 85 ± 15 | 84 ± 15 | 85 ± 15 | 0.36 |
|  |  |  |  |  |  |
| Heart rate, mean (SD), beats/min | 74 ± 15 | 75 ± 14 | 74 ± 16 | 80 ± 20 | 0.08 |
| Ischemia time, median (IQR), min | 164 (118 - 216) | 140 (96 - 244) | 173 (110 - 273) | 158 (111 - 288) | 0.24 |
| Single vessel disease – No. (%) | 65 (70) | 63 (69) | 63 (68) | 64 (70) | 0.99 |
| Culprit vessel – No (%) |  |  |  |  | 0.07 |
| LAD | 29 (31%) | 38 (41%) | 39 (42%) | 39 (42%) |  |
| LCX | 15 (16%) | 18 (20%) | 8 (9%) | 20 (22%) |  |
| RCA | 49 (53%) | 36 (39%) | 45 (49%) | 33 (36%) |  |
| Infarct-related artery TIMI flow – No. (%) |  |  |  |  |  |
| Pre-intervention grade |  |  |  |  | 0.26 |
| 0 | 43 (46) | 54 (59) | 59 (64) | 50 (54) |  |
| 1 | 7 (8) | 4 (4) | 7 (8) | 7 (8) |  |
| 2 | 21 (23) | 14 (15) | 9 (10) | 20 (22) |  |
| 3 | 22 (24) | 20 (22) | 17 (18) | 15 (16) |  |
| Post-intervention grade |  |  |  |  | 0.05 |
| 2 | 5 (5) | 6 (7) | 15 (16) | 8 (9) |  |
| 3 | 88 (95) | 86 (93) | 77 (84) | 84 (91) |  |
| Myocardial blush grade |  |  |  |  | 0.58 |
| 0 | 1 (1) | 3 (3) | 3 (3) | 3 (3) |  |
| 1 | 4 (4) | 5 (5) | 10 (11) | 10 (11) |  |
| 2 | 16 (17) | 21 (23) | 19 (21) | 18 (20) |  |
| 3 | 71 (77) | 63 (68) | 58 (64) | 61 (66) |  |
| Laboratory values at admission |  |  |  |  |  |
| CK, median (IQR), U/l | 122 (77 - 177) | 126 (86 - 171) | 136 (93 - 255) | 143 (89 - 343) | 0.038 |
| Myocardial band of CK, median (IQR), U/l | 14 (12 - 22) | 16 (13 - 21) | 16 (13 - 23) | 20 (13 - 55) | <0.001 |
| Troponin, median (IQR), ng/l | 37 (20 - 86) | 44 (22 - 89) | 52 (27 - 165) | 68 (27 - 315) | 0.007 |
| Creatinine, median (IQR), umol/l | 71 (61 - 80) | 71 (64 - 82) | 71 (60 - 81) | 76 (64 - 85) | 0.44 |
| NT-proBNP, median (IQR), ng/l | 66 (35 - 138) | 80 (32 - 166) | 86 (42 - 176) | 112 (52 - 487) | 0.004 |
| Glucose (median (IQR), mmol/l | 7.7 (6.7 - 8.5) | 8.6 (7.3 - 9.7) | 8.7 (7.2 - 9.7) | 8.8 (7.5 - 10.4) | <0.001 |
| HbA1c, median (IQR), % | 5.8 (5.6 - 6) | 5.8 (5.6 - 6) | 5.8 (5.6 - 6.1) | 5.8 (5.6 - 6.1) | 0.95 |
| Blood count and biochemistry |  |  |  |  |  |
| Leucocytes (10e9/l) | 11 (8.3 - 13.6) | 10.5 (8.4 - 13) | 11.4 (9.0 - 13.8) | 11.4 (9.5 - 13.9) | 0.14 |
| Thrombocytes (10e9/l) | 234 (206 - 267) | 224 (195 - 260) | 243 (210 - 282) | 245 (210 - 273) | 0.099 |
| Neutrophils (10e9/l) | 8.2 (5.5 - 9.9) | 7.18 (4.96 - 9.04) | 8.5 (5.7 - 9.8) | 7.9 (5.9 - 10.4) | 0.37 |
| Lymphocytes (10e9/l) | 1.8 (1.3 - 2.4) | 2.1 (1.5 - 2.6) | 1.8 (1.4 - 2.5) | 2.1 (1.5 - 2.9) | 0.16 |
| N/L ratio | 3.9 (2.9 - 6.6) | 3.3 (1.9 - 5.4) | 4.2 (2.8 - 6.3) | 3.4 (2.1 - 6.4) | 0.26 |
| hsCRP (mg/l) | 1.6 (0.6 – 3) | 1.9 (0.9 - 3.1) | 2.3 (1.1 - 4.2) | 3.8 (1.6 - 7.6) | <0.001 |

Data are expressed as mean ± standard deviation (SD), median (interquartile range (IQR)), or as number (%). BMI = Body Mass Index; TIMI = Thrombolysis in Myocardial Infarction; CK = creatine kinase; NT-proBNP = N-terminal pro brain natriuretic peptide; HbA1c = glycated hemoglobin; N/L = neutrophil/lymphocyte.

Supplementary table 2. Baseline characteristics per sIL-6R quartile

| **Characteristics** |  | **Q1 (n = 92)** | **Q2 (n = 91)** | **Q3 (n = 92)** | **Q4 (n = 91)** | **P-value** |
| --- | --- | --- | --- | --- | --- | --- |
| sIL-6R levels (ng/ml) |  | 52180 (37164 - 71582) | 50730 (36787 - 69125) | 51170 (37175 - 69009) | 52241 (37920 - 68547 | <0.001 |
| Age, mean (SD), years |  | 58.3 ± 10.6 | 60.4 ± 11.1 | 58.3 ± 11.7 | 58.2 ± 12.9 | 0.52 |
| Female sex - No. (%) |  | 23 (25) | 23 (25) | 25 (27) | 23 (21) | 0.94 |
| BMI, mean (SD), kg/m^2^ |  | 26.9 ± 5.0 | 26.3 ± 3.1 | 27.1 ± 3.7 | 27.6 ± 3.4 | 0.17 |
| Hypertension |  | 30 (33) | 27 (30) | 26 (28) | 25 (27) | 0.88 |
| Dyslipidemia |  | 59 (64) | 55 (60) | 60 (65) | 54 (59) | 0.82 |
| Current smoking |  | 58 (63) | 45 (49) | 56 (61) | 45 (49) | 0.12 |
| Stroke |  | 1 (1) | 0 (0) | 0 (0) | 2 (2) | 0.29 |
| Peripheral artery disease |  | 0 (0) | 0 (0) | 0 (0) | 0 (0) | 1.00 |
| Previous PCI |  | 1 (1) | 1 (1) | 1 (1) | 1 (1) | 1.00 |
| Blood pressure, mean (SD) mmHg |  |  |  |  |  |  |
| Systolic |  | 134 ± 22 | 137 ± 27 | 131 ± 25 | 134 ± 20 | 0.29 |
| Diastolic |  | 85 ± 14 | 86 ± 16 | 82 ± 16 | 84 ± 12 | 0.20 |
| Heart rate, mean (SD), beats/min |  | 79 ± 13 | 76 ± 20 | 75 ± 17 | 72 ± 14 | 0.06 |
| Ischemia time, median (IQR), min |  | 175.5 (107.5 - 260.5) | 161 (108 - 266) | 148 (109.5 - 217) | 161 (105 - 254) | 0.78 |
| Single vessel disease - No. (%) |  | 64 (70) | 69 (76) | 65 (71) | 56 (62) | 0.22 |
| Culprit vessel - No. (%) |  |  |  |  |  | 0.22 |
| LAD |  | 45 (49) | 39 (43) | 28 (30) | 32 (35) |  |
| LCX |  | 12 (13) | 13 (14) | 19 (21) | 17 (19) |  |
| RCA |  | 35 (38) | 39 (43) | 45 (49) | 42 (46) |  |
| Infarct-related artery TIMI flow – No. (%) |  |  |  |  |  |  |
| Pre-intervention grade |  |  |  |  |  | 0.48 |
|  |  | 56 (61) | 56 (62) | 49 (53) | 44 (48) |  |
|  |  | 7 (8) | 8 (9) | 4 (4) | 6 (7) |  |
|  |  | 12 (13) | 13 (14) | 20 (22) | 18 (20) |  |
|  |  | 17 (18) | 14 (15) | 19 (21) | 23 (25) |  |
| Post-intervention grade |  |  |  |  |  | 0.88 |
|  |  | 7 (8) | 10 (11) | 9 (10) | 8 (9) |  |
|  |  | 85 (92) | 81 (89) | 83 (90) | 83 (91) |  |
| Myocardial blush grade |  |  |  |  |  |  |
|  |  | 2 (2) | 3 (3) | 3 (3) | 2 (2) | 0.99 |
|  |  | 7 (8) | 9 (10) | 5 (6) | 8 (9) |  |
|  |  | 21 (23) | 17 (19) | 18 (20) | 17 (19) |  |
|  |  | 62 (67) | 62 (68) | 63 (71) | 64 (70) |  |
| Laboratory values at admission |  |  |  |  |  |  |
| CK, median (IQR), U/l |  | 139 (83 - 255) | 129 (86 - 192) | 122 (85 - 208) | 126 (85 - 198) | 0.89 |
| Myocardial band of CK, median (IQR), U/l |  | 18 (13 - 42.5) | 16 (13 - 25) | 15 (12 - 22.5) | 16 (12 - 23) | 0.09 |
| Troponin, median (IQR), ng/l |  | 42 (21 - 174) | 60 (29 - 133) | 40 (16 - 135) | 50 (29 - 115) | 0.34 |
| Creatinine, median (IQR), umol/l |  | 70 (59 - 80.5) | 72 (60 - 82) | 76 (65.5 - 85) | 71 (64 - 82) | 0.19 |
| NT-proBNP, median (IQR), ng/l |  | 116 (41 - 277) | 91 (54 - 233) | 65.5 (30 - 167) | 67 (35 - 170) | 0.03 |
| Glucose (median (IQR), mmol/l |  | 8.2 (6.5 - 9.7) | 8.4 (7.2 - 9.8) | 8.25 (7.05 - 9.2) | 8.2 (7 - 9.4) | 0.82 |
| HbA1c, median (IQR), % |  | 5.8 (5.6 - 6) | 5.8 (5.6 - 6.1) | 5.7 (5.6 - 6) | 5.9 (5.5 - 6.1) | 0.47 |
| Blood count and biochemistry |  |  |  |  |  |  |
| Leucocytes (10e9/l) |  | 11.4 (8.5 - 14.05) | 11.4 (8.9 - 13.7) | 11 (9.65 - 13.6) | 10.2 (8.4 - 12.9) | 0.17 |
| Thrombocytes (10e9/l) |  | 233 (206 - 267) | 239 (200 - 274) | 243 (215 - 278) | 220 (188 - 266) | 0.06 |
| Neutrophils (10e9/l) |  | 7.9 (5.5 - 11.2) | 7.6 (5.3 - 10.6) | 8.3 (6.0 - 9.8) | 6.9 (5.2 - 9.0) | 0.13 |
| Lymphocytes (10e9/l) |  | 1.97 (1.5 - 2.4) | 2.0 (1.5 - 2.7) | 2.0 (1.4 - 2.7) | 2.0 (1.4 - 2.6) | 0.98 |
| N/L ratio |  | 3.7 (2.6 - 6.7) | 3.8 (2.3 - 6.1) | 4.0 (2.9 - 6.9) | 3.3 (2.2 - 5.6) | 0.48 |
| hsCRP (mg/l) |  | 2.55 (1.4 - 5) | 2.4 (1.1 - 3.7) | 1.8 (.8 - 3.5) | 1.7 (.9 - 4.1) | 0.28 |

Data are expressed as mean ± standard deviation (SD), median (interquartile range (IQR)), or as number (%). BMI = Body Mass Index; TIMI = Thrombolysis in Myocardial Infarction; CK = creatine kinase; NT-proBNP = N-terminal pro brain natriuretic peptide; HbA1c = glycated hemoglobin; N/L = neutrophil/lymphocyte.

Supplementary table 3. Baseline characteristics per sgp130 quartile

| **Characteristics** | **Q1 ( n = 88)** | **Q2 (n = 85)** | **Q3 (n = 87)** | **Q4 (n = 86)** | **P-value** |
| --- | --- | --- | --- | --- | --- |
| sgp130 levels (ng/ml) | 235 (195 - 259) | 311 (292 - 320) | 364 (345 - 381) | 453.5 (425 - 513) | <0.001 |
| Age, mean (SD), years | 59.0 ± 11.4 | 60.6 ± 11.2 | 57.9 ± 12.7 | 59.0 ± 11.0 | 0.50 |
| Female sex - No. (%) | 28 (32) | 28 (33) | 19 (22) | 14 (16) | 0.03 |
| BMI, mean (SD), kg/m^2^ | 26.8 ± 3.8 | 26.8 ± 4.2 | 26.8 ± 4.0 | 27.4 ± 3.6 | 0.74 |
| Cardiovascular related history – No. (%) |  |  |  |  |  |
| Hypertension | 24 (27) | 26 (31) | 27 (31) | 27 (31) | 0.93 |
| Dyslipidemia | 57 (65) | 50 (59) | 51 (59) | 56 (65) | 0.70 |
| Current smoking | 46 (52) | 46 (54) | 51 (59) | 47 (55) | 0.86 |
| Stroke | 0 (0) | 0 (0) | 1 (1) | 2 (2) | 0.29 |
| Peripheral artery disease | 0 (0) | 0 (0) | 0 (0) | 0 (0) | 1.00 |
| Previous PCI | 1 (1) | 0 (0) | 2 (2) | 1 (1) | 0.56 |
| Blood pressure, mean (SD) mmHg |  |  |  |  |  |
| Systolic | 133 ± 22 | 131 ± 24 | 135 ± 23 | 138 ± 25 | 0.21 |
| Diastolic | 84 ± 13 | 81 ± 14 | 86 ± 15 | 87 ± 16 | 0.02 |
| Heart rate, mean (SD), beats/min | 74 ± 13 | 77 ± 18 | 77 ± 17 | 77 ± 16 | 0.51 |
| Ischemia time, median (IQR), min | 163 (108 - 241) | 153 (109 - 236) | 161 (96 - 304) | 160 (120 - 229) | 0.99 |
| Single vessel disease – No. (%) | 57 (65) | 62 (73) | 61 (70) | 58 (67) | 0.69 |
| Culprit vessel – No (%) |  |  |  |  |  |
| LAD | 33 (38) | 38 (45) | 32 (37) | 36 (42) | 0.74 |
| LCX | 11 (13) | 12 (14) | 15 (17) | 16 (19) |  |
| RCA | 44 (50) | 35 (41) | 40 (46) | 34 (40) |  |
| Infarct-related artery TIMI flow – No. (%) |  |  |  |  |  |
| Pre-intervention grade |  |  |  |  |  |
| 0 | 54 (61) | 47 (55) | 48 (55) | 48 (56) | 0.15 |
| 1 | 4 (5) | 3 (4) | 11 (13) | 4 (5) |  |
| 2 | 14 (16) | 18 (21) | 17 (20) | 12 (14) |  |
| 3 | 16 (18) | 17 (20) | 11 (13) | 22 (26) |  |
| Post-intervention grade |  |  |  |  |  |
| 2 | 6 (7) | 11 (13) | 10 (11) | 6 (7) | 0.40 |
| 3 | 82 (93) | 74 (87) | 77 (89) | 80 (93) |  |
| Myocardial blush grade |  |  |  |  |  |
| 0 | 1 (1) | 5 (6) | 3 (4) | 1 (1) | 0.12 |
| 1 | 10 (11) | 8 (9) | 8 (10) | 3 (3) |  |
| 2 | 12 (14) | 22 (26) | 20 (24) | 18 (21) |  |
| 3 | 65 (74) | 50 (59) | 53 (63) | 64 (74) |  |
| Laboratory values at admission |  |  |  |  |  |
| CK, median (IQR), U/l | 131 (86 - 241) | 108 (81 - 176) | 143 (100 - 213) | 140 (89 - 260) | 0.12 |
| Myocardial band of CK, median (IQR), U/l | 15 (12 - 24) | 17 (13 - 25) | 18 (13 - 31) | 16 (14 - 23) | 0.76 |
| Troponin, median (IQR), ng/l | 42 (28 - 137) | 60 (26 - 130) | 49 (20 - 158) | 48 (25 - 134) | 0.97 |
| Creatinine, median (IQR), umol/l | 70 (60 - 79) | 74 (60 - 81) | 70 (62 - 81) | 77 (65 - 85) | 0.12 |
| NT-proBNP, median (IQR), ng/l | 88 (37 - 230) | 80 (37 - 200) | 64 (41 - 143) | 105 (51 - 266) | 0.41 |
| Glucose (median (IQR), mmol/l | 8.2 (7.3 - 9.9) | 9 (7.6 - 10.5) | 8.1 (6.9 - 9) | 8.2 (7.1 - 9.3) | 0.02 |
| HbA1c, median (IQR), % | 5.7 (5.6 - 6) | 5.8 (5.6 - 6) | 5.8 (5.6 - 6.1) | 5.8 (5.6 - 6.1) | 0.70 |
| Blood count and biochemistry |  |  |  |  |  |
| Leucocytes (10e9/l) | 11.1 (8.9 - 14.0) | 11.6 (8.5 - 14.1) | 11.1 (8.9 - 13.4) | 10.7 (8.6 - 13.8) | 0.88 |
| Thrombocytes (10e9/l) | 245 (212 - 276) | 242 (209 - 276) | 227 (187 - 270) | 231 (204 - 262) | 0.15 |
| Neutrophils (10e9/l) | 7.5 (5.6 - 9.8) | 8.2 (5.0 - 9.8) | 7.6 (5.7 - 10.1) | 8.1 (5.3 - 10.8) | 0.82 |
| Lymphocytes (10e9/l) | 1.9 (1.4 - 2.5) | 2.1 (1.5 - 2.9) | 2.0 (1.6 - 2.6) | 1.9 (1.3 - 2.4) | 0.12 |
| N/L ratio | 3.6 (2.8 - 5.8) | 3.3 (2.1 - 5.1) | 3.4 (2.4 - 6.4) | 4.7 (2.4 - 7.4) | 0.11 |
| hsCRP (mg/l) | 2 (.8 - 3.8) | 2.7 (1.4 - 5.1) | 2.6 (1.3 - 5.1) | 1.7 (.8 - 3.9) | 0.09 |

Data are expressed as mean ± standard deviation (SD), median (interquartile range (IQR)), or as number (%). BMI = Body Mass Index; TIMI = Thrombolysis in Myocardial Infarction; CK = creatine kinase; NT-proBNP = N-terminal pro brain natriuretic peptide; HbA1c = glycated hemoglobin; N/L = neutrophil/lymphocyte.

Supplementary table 4. Number of samples available for measurements of IL-6, sIL-6R and sgp130 at the different time points

|  | **IL-6** | **sIL-6R** | **sgp130** |
| --- | --- | --- | --- |
| **Baseline** | 369 | 366 | 346 |
| **24 hours** | 341 | 342 | 316 |
| **2 weeks** | 325 | 329 | 309 |
| **7 weeks** | 316 | 319 | 297 |
| **4 months** | 313 | 314 | 293 |
| **1 year** | 262 | 268 | 252 |

IL-6 = interleukin 6; sIL-6R = soluble interleukin 6 receptor; sgp130 = soluble glycoprotein 130

Supplementary table 5. Associations between IL-6, sIL-6R and sgp130 and cardiac markers at baseline. * = p<0.005, ^#^ = p<0.05.

|  | **Troponin T** | **CK** | **CK-MB** | **NT-proBNP** |
| --- | --- | --- | --- | --- |
| **IL-6** | 0.19* | 0.19* | 0.22* | 0.22* |
| **sIL-6R** | -0.02 | -0.02 | -0.08 | -0.12^#^ |
| **sgp130** | 0 | 0 | 0.03 | 0.03 |
