# Supplementary figures and images for: Plasma interleukin 6 levels are associated with cardiac function after ST-elevation myocardial infarction
Source: Clin Res Cardiol. 2018 Oct 26;108(6):612–21. doi: 10.1007/s00392-018-1387-z (PMC6529378; doi:10.1007/s00392-018-1387-z)

**A**

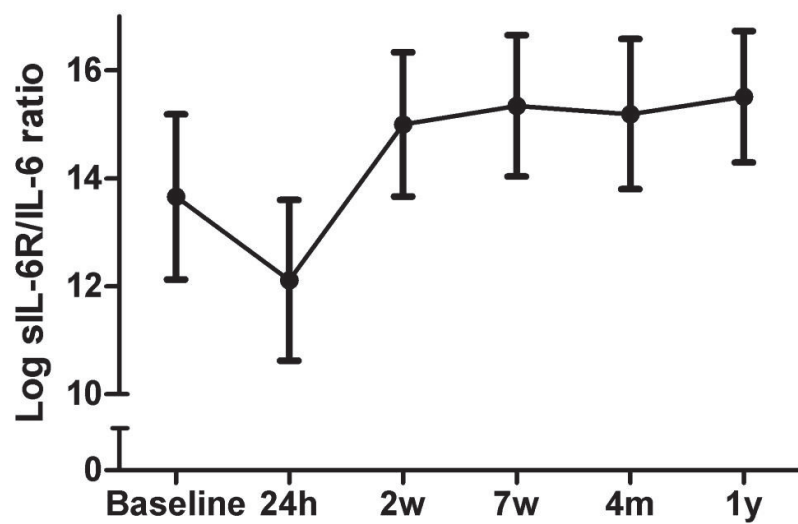

**B**

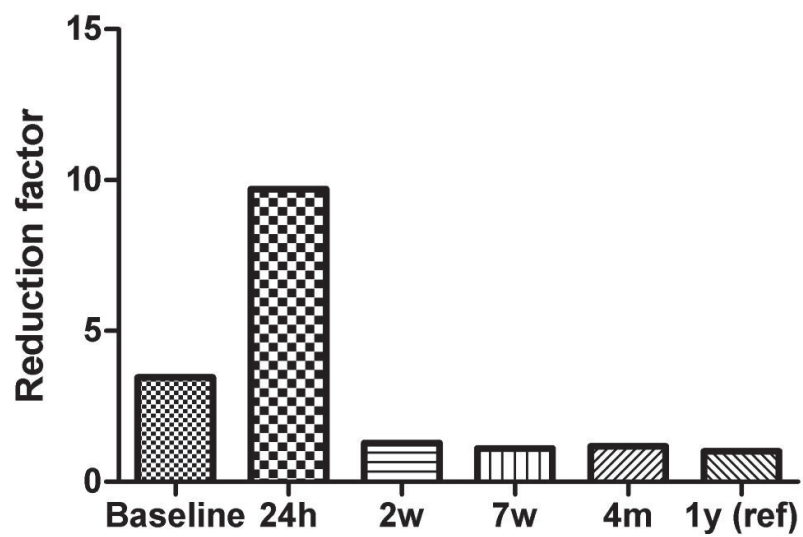

Supplementary Figure 1

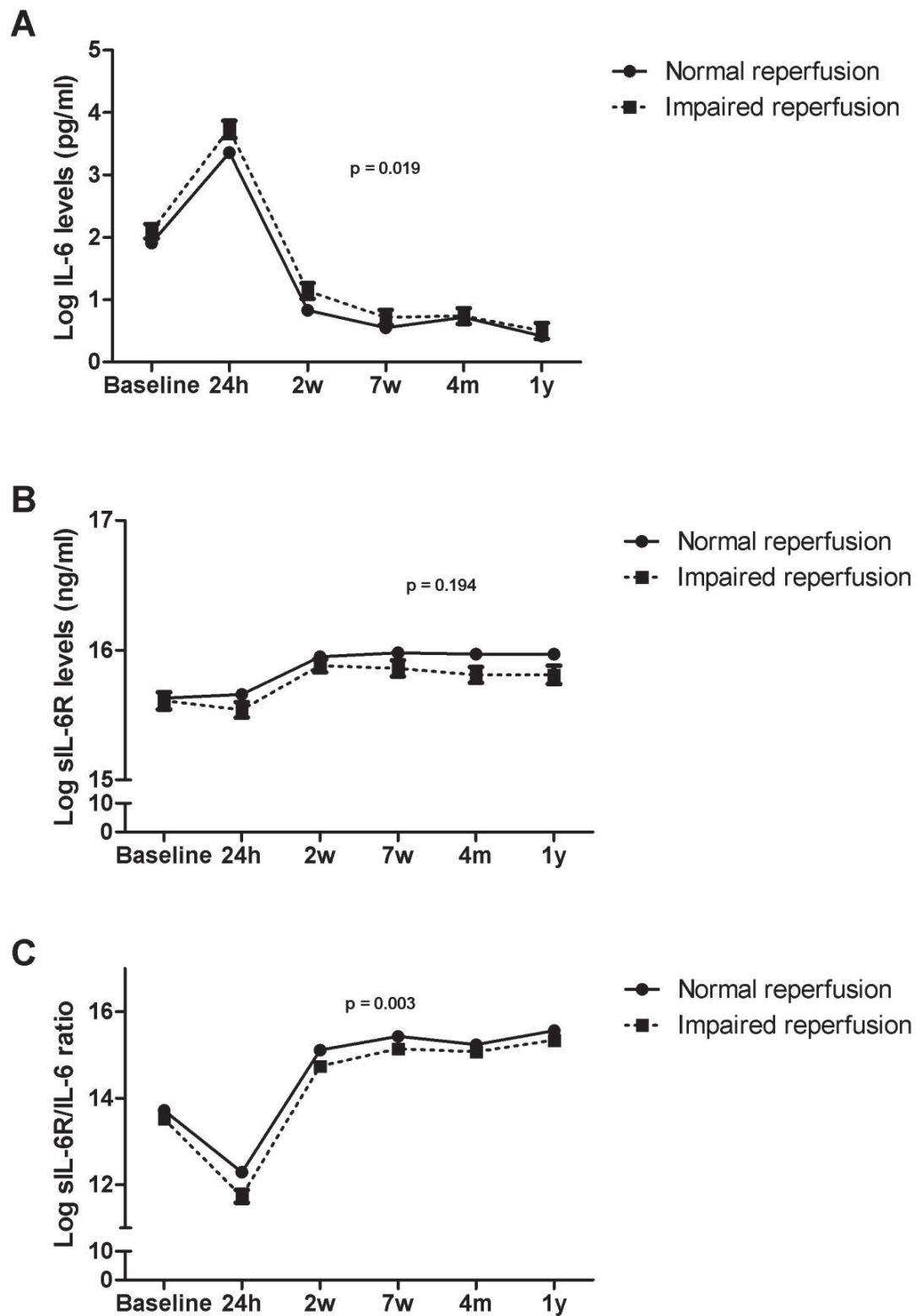

Supplementary Figure 2

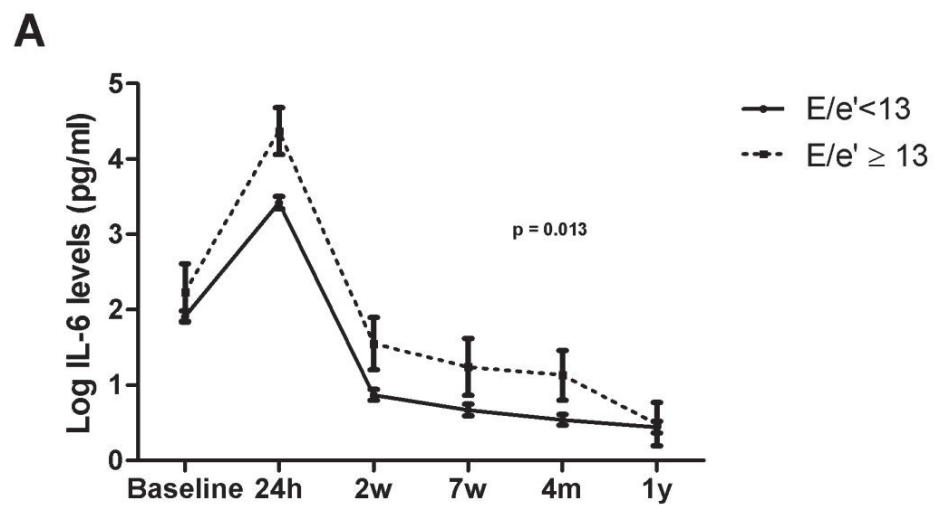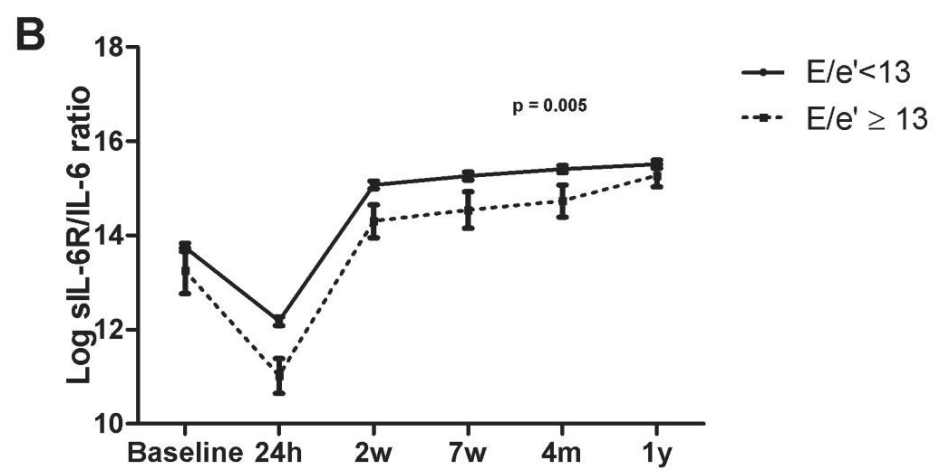

Supplementary Figure 3

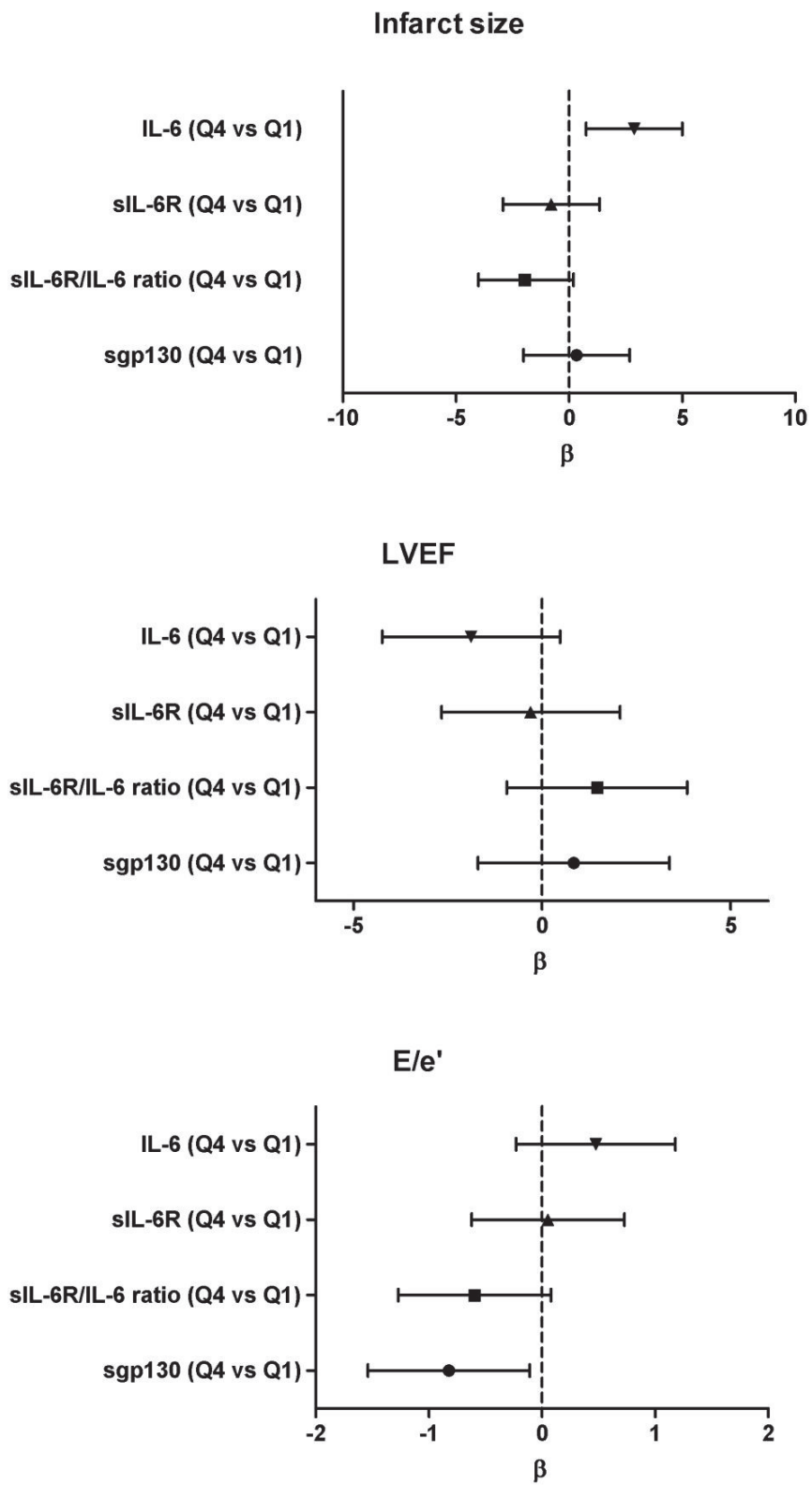

Supplementary Figure 4

Supplement: Supplementary file 2 — Supplementary Figure 1. Log sIL-6R/IL-6 ratio (mean and SD) (A). We divided log sIL-6R levels by IL-6 levels to be able to show a clear and understandable figure. Reduction factor of log sIL-6R/IL-6 ratio using log sIL-6R/IL-6 ratio at 1 year as a reference (B). Supplementary Figure 2. Log hs-CRP levels in the total STEMI population (A) and in STEMI patients with LVEF ≥ 50%, LVEF 40 – 49%, LVEF < 40%. Levels are depicted in mean and SEM. P values for trend are shown. Supplementary Figure 3. Log IL-6 (A), log sIL-6R (B), and log sIL-6R/IL-6 ratio (C) levels in STEMI patients with normal (MBG 3) and impaired reperfusion (MBG 0-2). Levels are depicted in mean and SEM (IL-6 and sIL-6R) or mean and SD (sIL-6R/IL-6 ratio). p values for trend are shown. Supplementary Figure 4. Log IL-6 (A), log sIL-6R/IL-6 (B) levels in STEMI patients with normal E/e’ (<13) and elevated E/e’ (≥ 13). Levels are depicted in mean and SEM. p values for trend are shown. Supplementary Figure 5. Associations between members of the interleukin-6 signaling cascade measured in STEMI patients at baseline and infarct size, LVEF, and E/eˈ ratio measured at 4 months, depicted as β and 95% CIs obtained from linear regression models. 95% CI = 95% Confidence Interval; IL-6 = interleukin 6; sIL-6R = soluble interleukin 6 receptor; sgp130 = soluble glycoprotein 130; Q1 = lowest quartile; Q4 = highest quartile; STEMI = ST-elevation myocardial infarction; LVEF = left ventricular ejection fraction. Multivariate analysis on infarct size: adjusted for age, sex, BMI, hypercholesterolemia, TIMI (pre- and post-intervention), MBG. Multivariate analysis on LVEF: adjusted for age, sex, hypercholesterolemia, TIMI (pre- and post-intervention), ischemic time. Multivariate analysis on E/eˈ: adjusted for age, sex, BMI, heart rate, hypertension, smoking (PDF 743 KB) [file 392_2018_1387_MOESM2_ESM.pdf]
